# The shadows of a ghost: a survey of canine leishmaniasis in Presidente Prudente and its spatial dispersion in the western region of São Paulo state, an emerging focus of visceral leishmaniasis in Brazil

**DOI:** 10.1186/s12917-015-0583-6

**Published:** 2015-10-26

**Authors:** Lourdes Aparecida Zampieri D’Andrea, Elivelton da Silva Fonseca, Luiz Euribel Prestes-Carneiro, Raul Borges Guimarães, Renata Corrêa Yamashita, Célio Nereu Soares, Roberto Mitsuyoshi Hiramoto, José Eduardo Tolezano

**Affiliations:** Center for Biomedical Sciences and Regional Laboratory, Adolfo Lutz Institute, Avenida Coronel Marcondes, 2357, Presidente Prudente, São Paulo Brazil; Health and Geography Laboratory, Faculty of Science and Technology, Universidade Estadual Paulista, Rua Roberto Simonsen, 305, Presidente Prudente, São Paulo Brazil; Immunology and Infectious Diseases Department, Oeste Paulista University, Rua José Bongiovani 700, Presidente Prudente, São Paulo Brazil; Center of Zoonosis Control, Rua Presidente Castelo Branco 93, Presidente Prudente, São Paulo Brazil; Center for Parasitology and Mycology, Adolfo Lutz Institute, Av. Dr. Arnaldo, 355, São Paulo, Brazil

**Keywords:** Canine leishmaniasis, *Ehrlichia canis*, *Leishmania infantum*

## Abstract

**Background:**

Visceral leishmaniasis is an emerging zoonosis and its geographic distribution is restricted to tropical and temperate regions. Most of the individuals infected in Latin America are in Brazil. Despite the control measures that have been adopted, the disease is spreading throughout new regions of the country. Domestic dogs are involved in the transmission cycle and are considered to be the main epidemiologic reservoir of *Leishmania infantum* (syn. *L. chagasi*). Our aim was to determine the prevalence of canine leishmaniasis (CL) and *Ehrlichiosis* infection in Presidente Prudente as well as the spatial dispersion of the disease in the western region of São Paulo state.

**Methods:**

Dogs underwent clinical examination and symptoms related to CL were recorded. Anti**-***Leishmania* antibodies were detected using ELISA, rK39-immunocromatographic tests (DPP), and an indirect fluorescent antibody test (IFAT). Anti-*E. canis* antibodies were detected by IFAT. A follow-up was conducted in dogs that were positive in the ELISA at the baseline study. Data on the spatial distribution of *L. longipalpis* and CL in São Paulo state were obtained from Brazilian public health agencies.

**Results:**

Serum samples from 4547 dogs were analyzed. The seroprevalence of CL was 11.2 % by ELISA and 4.5 % by IFAT. In the follow-up, seroprevalence was 32.9 % by ELISA, 15.3 % by IFAT, 11.8 % by DPP test, and 66.5 % for *E. canis*. There was a significant positive association between *Leishmania* and *E. canis* infection (*P* < 0.0001). In the follow-up, clinical examinations revealed symptoms compatible with CL in 33.5 % of the dogs. *L. longipalpis* was found in 24 and CL in 15 counties of the Presidente Prudente mesoregion. The dispersion route followed the west frontier of São Paulo state toward Paraná state.

**Conclusions:**

Low CL and high ehrlichiosis prevalence rates were found in Presidente Prudente city. This emerging focus of CL is moving through the western region of São Paulo state toward the border of Paraná state. Integrated actions to fight the vector, parasites, infected dogs, and humans are needed to monitor the disease and implement strategies for epidemiologic control.

## Background

Visceral leishmaniasis (VL) is an emerging zoonosis of great significance to public health; its geographic distribution is restricted to tropical and temperate regions [1]. About 90 % of the individuals infected with VL in Latin America are in Brazil. Zoonotic VL is a potentially fatal disease and domestic dogs are considered the main epidemiologic reservoir of *L. infantum* (syn. *L. chagasi*) due to intense skin and viscera parasitism. It is believed that some infected dogs do not develop clinical signs and remain asymptomatic for inconsistent periods of time [1, 2]. To control the spread of the disease, the Brazilian Ministry of Health created the Visceral Leishmaniasis Control and Surveillance Program (VLCSP), which is in charge of measures throughout the country, such as early diagnosis and treatment of human VL, identification and euthanasia of seropositive dogs, control of insect vectors, and health education [2]. Diagnosis of canine leishmaniasis (CL) is usually carried out using serologic enzyme-linked immunosorbent assay (ELISA), immunochromatographic tests, indirect fluorescent antibody test (IFAT), or molecular tests [1, 2]. *Ehrlichia canis*, a rickettsia microorganism transmitted by ticks, is one of the major vector-borne diseases in dogs with a worldwide distribution and is directly involved in the infection and spread of CL [3].

Despite the measures adopted by the VLCSP to control VL [2], it is suspected that the disease is spreading fast throughout the western region of São Paulo state [4–6]. From 1997, when *Lutzomyia longipalpis* was demonstrated in the municipality of Araçatuba in the neighboring region of Presidente Prudente, up until 2014, 93.3 % of cases of canine or human transmission of VL in São Paulo occurred in the western region [7]. Our aim was to determine the prevalence of CL using different methods and the prevalence of *E. canis* infection in Presidente Prudente as well as the spatial dispersion of the disease in western São Paulo state, Brazil.

## Methods

### Ethical considerations

Canine samples were collected with informed consent from dog owners and the project was approved by the Ethics Committee on the Use of Animals of Adolfo Lutz Institute/Pasteur Institute (ECUA, ALI/Pasteur), license number 07/12.

### Study design

A cross-sectional study was conducted between January 2010 and July 2011in the city of Presidente Prudente, in the western region of São Paulo state, Brazil (coordinates: latitude 22°07'32"S and longitude 51°23'20" W) and in the district of Montalvão. This city is a mid-sized urban center located 560 km from the state capital, São Paulo; according to the census by the Brazilian Institute of Geography and Statistics (IBGE) in 2012, the human population was 207,725 inhabitants. The municipality covers an area of 562,107 km^2^ and the urban area covers 16.56 km^2^. The district of Montalvão is located 12 km from Presidente Prudente city with a population of 2,229 and an urban area of 0.507 km^2^ and shares the same risk factors for CL (Fig. 2). The canine population was estimated to be 39,800 animals, with a ratio of 2 dogs per 10 ten inhabitants, according to the Center for Zoonosis Control (CZC) in Presidente Prudente, linked to the Municipal Secretary of Health. At the time of the study, the prevalence of human and canine leishmaniasis was not known in the municipality of Presidente Prudente. Hence, minor control actions of the VLCSP were in place. The region has a typical tropical climate with a dry winter and wet summer and an average annual temperature of 23.5 °C.

### Clinical examination and blood collection

The city of Presidente Prudente is distributed into 7 μ areas with regard to epidemiologic diseases, including dengue fever, canine VL, and human VL. In the first phase of this cross-sectional study, the canine selection took place in different regions of the city by passive demand and active searches conducted by the CZC. An active search was conducted when *L. longipalpis* was found in a particular area of the city or when a dog was confirmed to have CL. Immediately, all the households within a radius of 200 m were visited and the dogs investigated for CL. A passive search was conducted when a dogs’ owner found the symptoms described by the CZC suspicious for CL. The owners of the animals enrolled in the study were interviewed by a trained research team and a questionnaire was used to provide details including address, name, gender, age, race, housing conditions, origin of the animal (district of residence/other district). In the study, 4547 domiciled dogs of different ages and races underwent a veterinary examination by a team of 10 employees from the CZC and were classified as asymptomatic (absence of signs and symptoms of *Leishmania* spp. infection) or symptomatic. According to the Manual of Surveillance and Control of American Visceral Leishmaniasis in São Paulo state [8], the main clinical signs observed in symptomatic dogs included skin lesions consisting of areas of alopecia with eczematous desquamation mainly around the eyes, articulations, and skin folds, frequently followed by keratoconjunctivitis, onichogryphosis, generalized lymphadenopathy, and hepatosplenomegaly. Slight weight loss and anemia frequently occur.

A 5-mL blood sample was collected from each animal by venipuncture for serologic testing.

### Follow-up studies

A follow-up study was initiated 64.5 ± 2.05 days after the baseline study and a total of 170 dogs with positive ELISA were enrolled. During this period, the Brazilian Ministry of Health introduced the DPP rapid test for the diagnosis of CL. Because a limited number of DPP tests (200) were available, only 170 animals were enrolled in the follow-up. Furthermore, there was a strong suspicion of a cross-reaction between CL and the serologic diagnosis of ehrlichiosis. To study this question, the municipality acquired an *E. canis* IFAT reagent with 200 test samples. All dogs included in the follow-up underwent the same procedures as used in the baseline protocol plus *E. canis* IFAT.

### Antibody tests

The ELISA was used for the detection of anti-*Leishmania* IgG antibodies in the canine survey (EIE-Leishmaniose-Visceral-Canine-Bio-Manguinhos [EIE-LVC] kit; Bio-Manguinhos/Fiocruz, Ministry of Health, Rio de Janeiro, Brazil). The sensitivity of the EIE-LVC was previously determined to be 72 and 100 % and the specificity was 87.5 and 96.6 %, respectively [9, 10]. The ELISA-positive samples were later confirmed by IFAT (IFAT-Leishmaniose-Visceral-Canine-Bio-Manguinhos [IFAT-LVC] kit; Bio-Manguinhos/Fiocruz, Ministry of Health, Rio de Janeiro, Brazil), as recommended by the Brazilian Ministry of Health [2]. The sensitivity of the IFAT-LVC was previously determined to be 68.0 and 100 % and the specificity was 87.5 and 96.6 %, respectively [9, 10]. The rapid test for the detection of anti-*Leishmania* IgG antibodies was performed using a dual path platform (DPP) CL rapid test (Bio-Manguinhos/Fiocruz, Ministry of Health, Rio de Janeiro, Brazil), according to the manufacturer’s instructions. The test uses DPP recombinant protein K39 (rK39) antigen, a 39 amino acid sequence of the cloned specific kinase region of *L. infantum*, which is widely used in the diagnosis of CL [11]. For the immunochromatographic assay, rK39 had a sensitivity of 91.5 % and specificity of 94.7 % in a previous study [11].

The detection of *Ehrlichia canis* antibodies by IFAT was conducted according to the manufacturer’s instructions (VMRD, Inc., Pullman, WA, USA), and serum samples, diluted at 1:20, were considered positive. Previous results of serological tests analyzing IFAT ehrlichiosis showed 100 % sensitivity and 98.5 % specificity [12]. All dogs showing positive serology on ELISA and confirmed by IFAT were euthanized by the veterinarian in charge in accordance with the recommendations of the VLCSP program [2, 8].

### Geographic expansion of *L. longipalpis* and CL in São Paulo state

The state of São Paulo has 645 municipalities and the Health State Department is divided into 17 mesoregions (Fig. 3a). The geographic expansion of *L. longipalpis* (Fig. 3b) and CL (Fig. 3c) is based on the year in which the first case was detected. Data were obtained from the following Brazilian public health agencies: Supervision and Control of Epidemic (SUCEN); Center of Regional Laboratory of the Adolfo Lutz Institute of Presidente Prudente V (CRL-CBSALI-PPV); National System of Diseases Notification (SINAN), São Paulo Epidemiological Bulletin (BEPA), and Municipal Surveillance Epidemiology Center (MSEC). The databases for the maps were collected from the IBGE website. The maps showing the spatial distribution were made using the ArcGIS 10.2 platform (ESRI 2014). The concentration of positive tests in Presidente Prudente city and Montalvão district was based on Gaussian kernel. Kernel density creates an output raster surface with the density of points plotted in an area. The Gaussian function of kernel density is a bell-shaped function that falls off quickly toward positive and negative infinity.

### Statistical analysis

The proportion (95 % confidence limits) of seropositive dogs (seroprevalence) was estimated. The Fisher exact test was used to compare seroprevalence across clinical groups and agreement between serological tests was assessed using kappa analysis. The kappa values were interpreted as follows: <0, no agreement; 0–0.19, poor agreement; 0.20–0.39, fair agreement; 0.40–0.59, moderate agreement; 0.60–0.79, substantial agreement; 0.80–1.00, almost perfect agreement [13]. Significance was taken at 5 % (*P* < 0.05) for a double-tailed test.

## Results

**Fig. 1:**
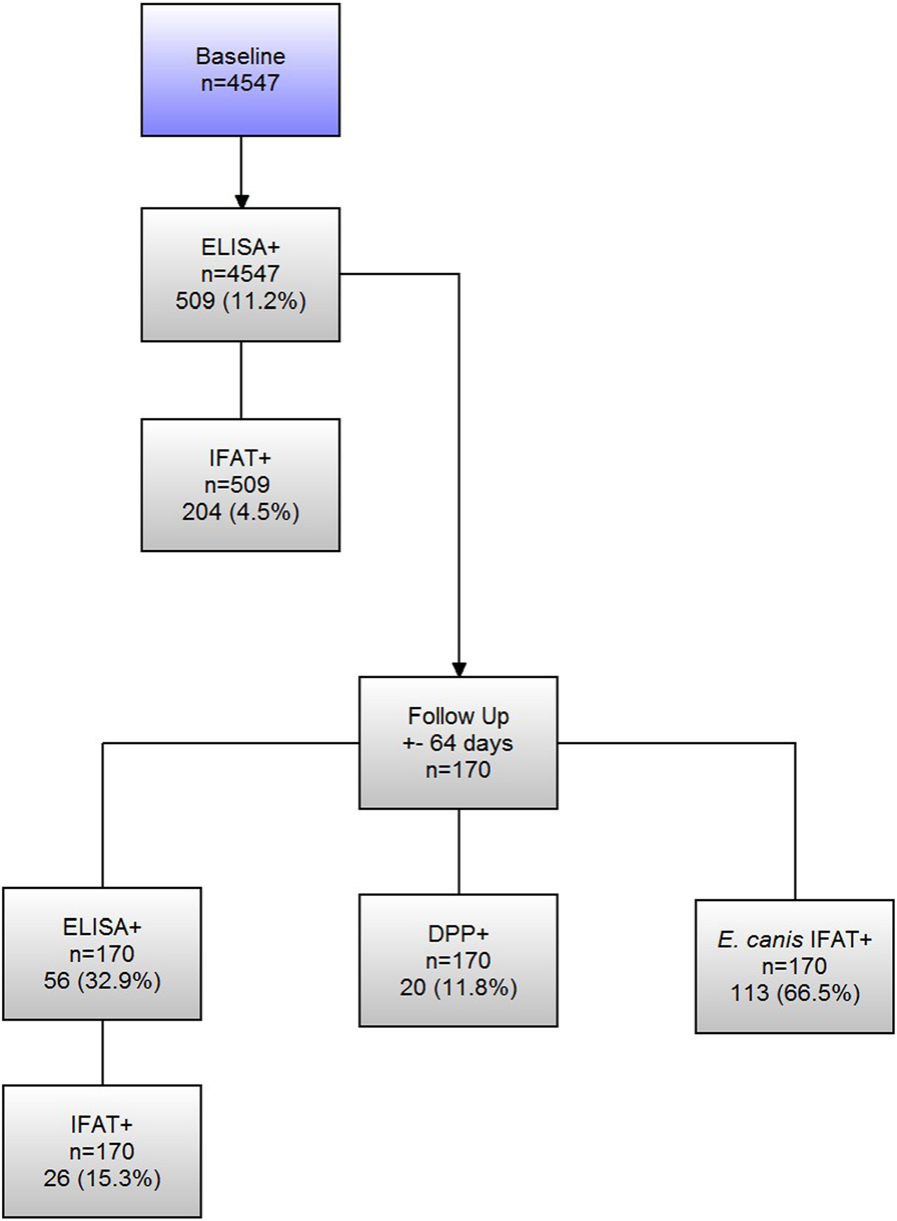
Seroprevalence of canine leishmaniasis and ehrlichiosis. Flowchart including baseline and follow-up data, the number of samples processed by each diagnostic test, and those testing positive

In the first phase of the study, 4547 dogs were enrolled from different areas of Presidente Prudente and Montalvão district, either by active or passive search. Clinical examinations were performed and of the serum samples submitted to the ELISA test for CL, 509 (11.2 %) were positive (Fig. 1): 204 samples were positive by IFAT, with a seroprevalence rate of 4.5 % (Fig. 1).

In the follow-up study of 170 animals, 56 were positive for the CL ELISA test and 20 were positive for the DPP test, with prevalence rates of 32.9 and 11.8 %, respectively, and 26 remained positive by IFAT with a prevalence rate of 15.3 %. With a seroprevalence rate of 66.5 %, 113 animals were found to be positive for *E. canis* infection (Fig. 1, follow-up). Forty-eight (28.2 %) of the animals showed leishmaniasis/ehrlichiosis co-infection by the ELISA/IFAT test and 10 (5.9 %) by the DPP/IFAT test. There was a significant positive association between *Leishmania* and *E. canis* infection (*P* < 0.0001). In the clinical examination of the follow-up cohort, 113 of 170 dogs (66.5 %) were classified as asymptomatic; 57 of 170 (33.5 %) presented one or more clinical signs connected with CL. In the follow-up, the kappa statistics for the results between ELISA and DPP resulted in fair agreement (κ = 0.222, 95 % CI, 0.04–0.2).

**Fig. 2:**
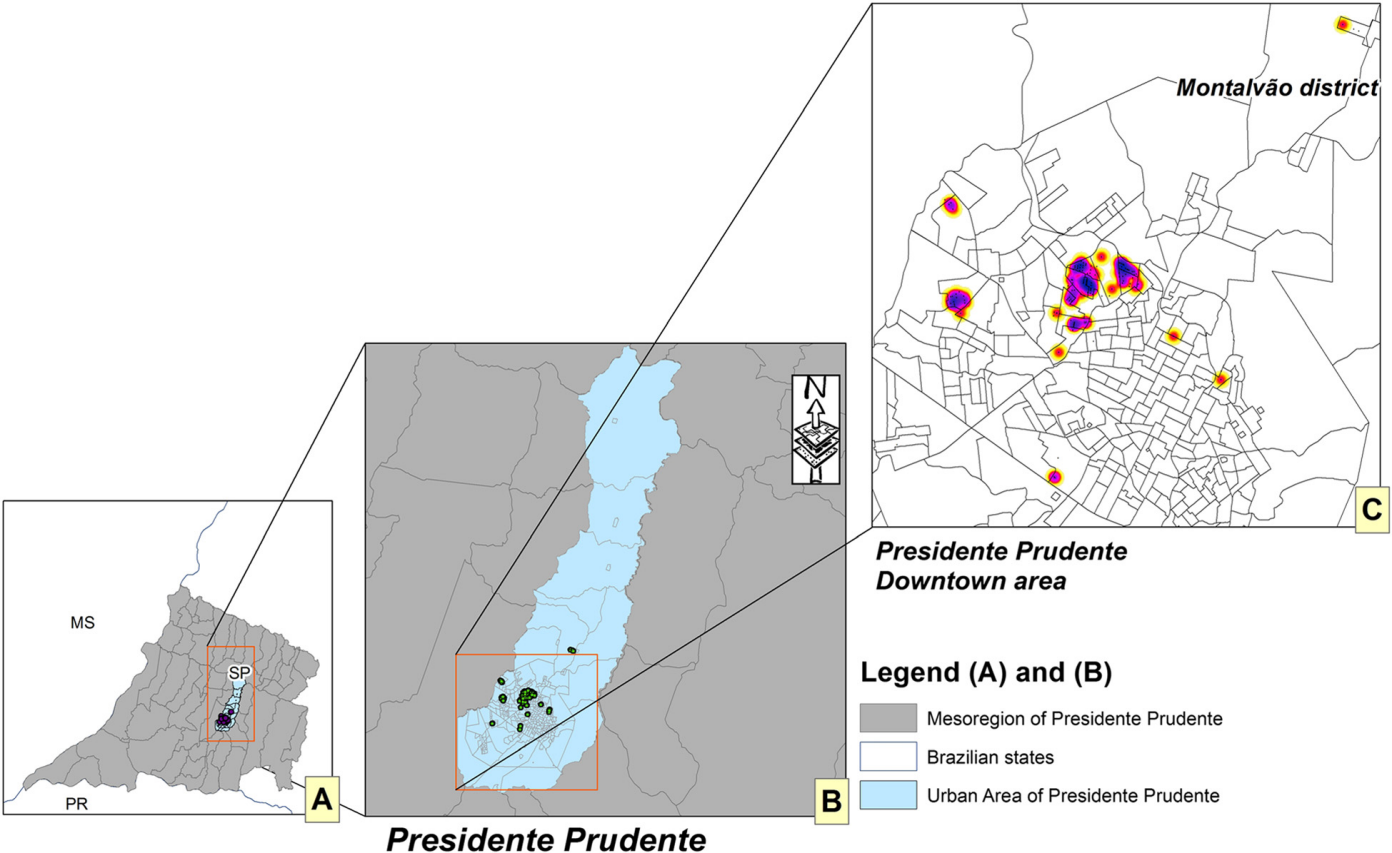
Canine leishmaniasis in the western region of São Paulo state. Spatial distribution of CL/IFAT in Presidente Prudente city and Montalvão district from January 2010 to July 2011. **a** SP, Western region of São Paulo state and bordering states; PR, Paraná; MS, Mato Grosso do Sul; **b** Presidente Prudente and bordering counties; **c** samples were plotted according to the owner’s address. Red, allochthonous CL seropositive dogs; yellow, autochthonous CL seropositive dogs. Source: IBGE, ALI PP V, and CZC of Presidente Prudente County

**Fig. 3:**
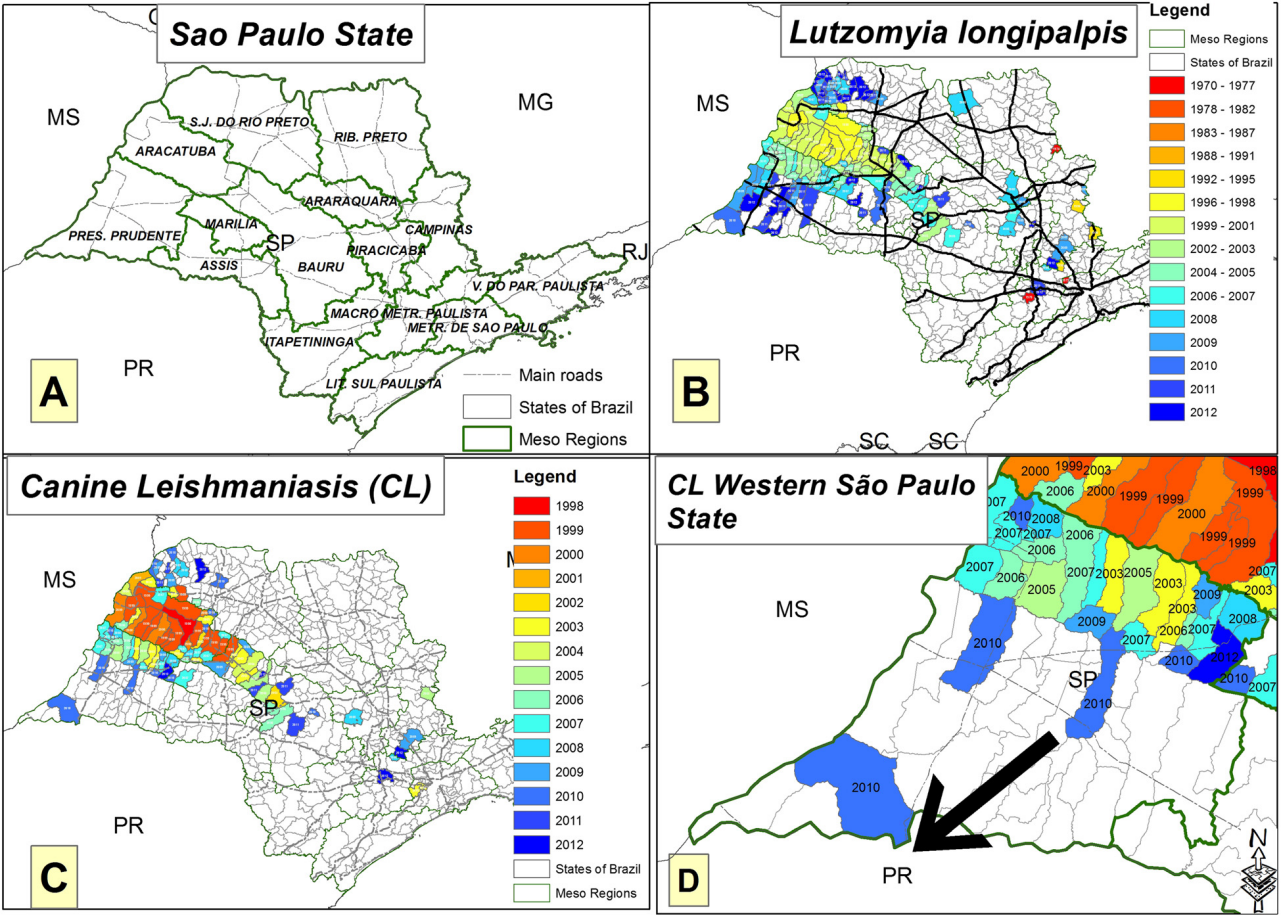
Distribution of *L. longipalpis* and canine leismaniasis in São Paulo state. The spatial distribution of (**a**) São Paulo state mesoregions (*green lines*) and main highways (*black lines*); (**b**) counties where *L. longipalpis* sandflies were found in São Paulo state; (**c**) counties of São Paulo state where dogs were diagnosed with CL for the first time; and (**d**) the spread of CL in counties of the western region of São Paulo state. The arrow shows the advancement of CL toward the border of the state of Paraná. The colors represent the evolution of CL events linked to the year. States bordering São Paulo: MS, Mato Grosso do Sul; PR, Paraná; MG, Minas Geraes; SC, Santa Catarina. Data were obtained from Brazilian public health agencies: Brazilian Institute of Geography and Statistics; Supervision and Control of Epidemic; Center Regional Laboratory Adolfo Lutz Institute of Presidente Prudente V; National System of Diseases Notification; and São Paulo Epidemiological Bulletin

Figure 2 shows the outcome for the IFAT serological confirmatory test according to the Brazilian Ministry of Health [2]. Seropositive animals were plotted according to the dog owner’s address at the time of collection. Based on the Gaussian kernel, it was possible to detect a concentration of positive tests in the downtown area, and the southwest and west regions of Presidente Prudente city and Montalvão district. The spatial distribution of vectors and CL throughout the study period in São Paulo state is shown in Fig. 3. *L. longipalpis* was found in 148 counties of São Paulo state (Fig. 3b); 32 of 45 counties (71.1 %) were located in Presidente Prudente’s mesoregion, of which eight are under investigation. CL was found in 102 counties (Fig. 3c); 15 of 45 counties (33.3 %) were located in Presidente Prudente’s mesoregion (Fig. 3d).

## Discussion

In this study carried out in Presidente Prudente city and in the district of Montalvão, the seroprevalence of CL was 11.2 and 4.5 % by ELISA and IFAT, respectively. When a follow-up was conducted, 32.9 % were positive on ELISA, 15.38 % on IFAT, and 11.8 % in the DPP test. As far as we know, this is the first description of autochthonous CL in this region. Furthermore, the disease is spreading to the border of Paraná state in the southern region of Brazil.

Until 1997, VL was known in the state of São Paulo only by imported cases. The first case of autochthonous CL was described in 1998 in Araçatuba, an area located about 160 km from Presidente Prudente, in the northwest region of the state [5, 6]. Thereafter, the infection spread throughout the administrative areas of Bauru, Marília, and São José do Rio Preto. Leishmaniasis has been detected in humans and dogs in the western region of São Paulo state, the first in the municipality of Dracena in 2005, considered by Brazil Health Ministry to be an area with a high transmission rate [4–6]. In São Paulo state, the vector and canine and human leishmaniasis are controlled by the Health Secretary of São Paulo state according to the Manual of Surveillance and Control of American Visceral Leishmaniasis [8]. Despite the measures adopted to control CL, the disease has spread quickly throughout the municipalities of the Dracena microregion. In recent years, like a ghost projecting its shadows, the disease has reached Presidente Prudente County, where canine VL was found in 2009 [5, 7].

In 2012, *L. longipalpis* and CL were found in some of the border counties of Paraná state, a region with few cases of VL [14]. With a large road network linking different regions, small towns and increasing levels of circulating people, animals and goods, the border area of both states is changing rapidly. Livestock breeding is being replaced by extensive agricultural mechanization of sugar cane plantations with aerial spraying of insecticides and herbicides. This new scenario may be slowing the burden of the disease in Paraná state. This scenario is not shared in Dracena and Presidente Prudente regions; they are notably less mechanized with predominantly areas of cattle pasture. There is a strong hypothesis that the route of expansion and dissemination of VL in the west and northwest region of São Paulo state originated in Bolivia [15]. The vector and parasite reached Brazil through Corumbá, on the western border of Mato Grosso do Sul state, moving on to Campo Grande, in the central region and many years after the disease was found in Três Lagoas, on the border of Andradina and Dracena, western São Paulo state. Later, it reached Bauru following the northwest railroad, Marechal Rondon highway, and recently, throughout the construction of the Bolivia-Brazil gas pipeline [6, 15].

The reasons why CL is spreading fast in the western region of São Paulo and in the direction of Paraná state are not well understood. With a tropical climate, dry winters and wet summers, the region consists of dozens of small towns and villages, and is historically one of the poorest regions in São Paulo state. It neighbors, Mato Grosso do Sul state and Araçatuba County, are well-known endemic foci of VL in Brazil. Certainly the lack of control of the canine population is one probable mechanism; packs of domestic and homeless dogs wander freely in urban areas, most of them without a zoonosis/vectors control service [4]. An overwhelming factor is the overlapping possibility of VL and cutaneous leishmaniasis throughout the region. Since 1950, outbreaks have occurred, mainly in the region of Pontal of Paranapanema [5]. In the present study, surprisingly, a significant number of infected dogs were found in the business district. Empty areas on the map do not mean the absence of infected animals; on the contrary, it means those places were not surveyed. Montalvão is a higher district of Presidente Prudente County. Linked by a highway, there is a daily flow of people for shopping, health care, work, and education services sharing the same risk factors for CL as Presidente Prudente.

The CL seroprevalence of 4.5 % is about 5-fold lower than the average rates of 23.8 % found in the counties of Dracena’s microregion, varying from 4.9 % in Monte Castelo to 29.2 and 30.0 % in Dracena and Santa Mercedes, respectively [4]. We suggest that low levels of parasites were circulating in Presidente Prudente city when the samples were obtained from dogs during the 2010–2011 period, only 1 year after the vector and CL were reported. Possibly, this is also the reason why high rates of discordance between the ELISA and IFAT methods were found. In endemic areas, with increasing rates of circulating parasites, the results between ELISA and IFAT tend to be closer [16–18]. Serological tests for leishmaniasis diagnosis, particularly in epidemiologic surveys of the active search, lack specificity and sensitivity because they are not completely purified and cross reactions may occur, mainly with canine blood-borne agents such as *E. canis* [17, 18]. In the city, the dispersion routes of CL occurred via allochthones dogs coming from surrounding endemic counties. Worldwide, in urban areas, the domestic dog is the main source of infection for the vector. They exhibit intense cutaneous parasitism, which allows easy infection from sandflies, and the maintenance of the epidemiologic cycle of transmission [17].

With an interval of 64.5 ± 2.05 days, 170 ELISA-positive dogs in the baseline study were re-tested and 56 (32.9 %) were positive in ELISA, 26 (15.3 %) were confirmed in IFAT, and 20 (11.8 %) were positive in the DPP test. An array of factors may be involved in the 3-fold reduction in the rate of ELISA-positive dogs in the follow-up compared with the baseline study. In a scenario of low endemicity, few parasites are in circulation, reducing the burden of infection; healthy animals may clear the parasite via a cell-mediated and humoral immune response resulting in low antibody concentration with negative or borderline titres at the follow-up. *E. canis*/IFAT showed a seroprevalence of 66.5 and 28.2 % in the animals with leishmaniasis/ehrlichiosis co-infection. Furthermore, significant levels of dogs infected with *E. canis* were found compared with CL, confirming the suspicions of the veterinarian team of the CZC in Presidente Prudente. Occurring in a wide geographic distribution, especially in tropical and subtropical areas, *E. canis* infection has a role in increasing susceptibility and making the clearance of *L. infantum* (syn. *L. chagasi*) difficult [3]. However, there are conflicting results on the role of *E. canis* in the cross-reaction of the serologic diagnosis of CL due to lack of specificity [3, 19, 20]. Our results demonstrate that canine ehrlichiosis was an important differential diagnosis of CL because of the prevalence of co-infected dogs. In areas of low CL endemicity, this aspect must be considered by clinicians; symptomatic dogs can be falsely diagnosed with CL and euthanized [19]. No data on the seroprevalence of ehrlichiosis were found in the region.

The association between DPP positive results and the presence of active clinical disease in both humans and dogs is well known [21]. In acute VL, the host may produce specific antibodies against replicating *Leishmania*. DPP antigens occur predominantly on amastigotes (the replicating form on human or dog hosts) and not in promastigotes [22]. In Brazil, until 2012, CL was diagnosed using IFAT to confirm positive cases detected by ELISA. After 2012, the DPP CL rapid test was introduced for screening with ELISA as a confirmatory test [23].

In the follow-up, 33.5 % of the animals were classified as symptomatic after clinical examination. These dogs did not originate from the passive or active search and they are not a representative sample for estimating CL.

## Conclusions

The data presented here allow us to conclude that low CL, suggesting low circulation of parasites and high prevalence rates of ehrlichiosis, were found in Presidente Prudente city. A cross-reaction between *L. infantum* (syn. *L. chagasi*) and *E. canis* antibodies may have occurred. CL is moving through the western counties of São Paulo state toward the border of Paraná state. Hence, implementation of integrated actions to fight the vector and the parasite, as well as the early diagnosis of dogs and humans, are needed for effective monitoring of the disease; in addition, strategies for epidemiologic control must be implemented.

## Abbreviations

ALI: Adolfo Lutz Institute
BEPA: São Paulo Epidemiological Bulletin
CL: Canine leishmaniasis
CRL-ALI-PPV: Center Regional Laboratory Adolfo Lutz Institute of Presidente Prudente V
CZC: Control Zoonosis Center
DPP: Dual path platform rK39-immunocromatographic tests
ECUA: Ethics Committee on the Use of Animals
EIE-LVC: EIE-Leishmaniose-Visceral-Canine-Bio-Manguinhos kit
ELISA: Enzyme-linked immunosorbent assay
IBGE: Brazilian Institute of Geography and Statistics
IFAT: Indirect fluorescent antibody test
IFAT-LVC: IFAT-Leishmaniose-Visceral-Canine-Bio-Manguinhos kit
MSEC: Municipal Surveillance Epidemiology Center
SINAN: National System of Diseases Notification
SUCEN: Supervision and Control of Endemic
VL: Visceral leishmaniasis
VLCSP: Visceral Leishmaniasis Control and Surveillance Program
